# Artificial intelligence for intimate apparel fit and comfort from technical evidence to business governance

**DOI:** 10.3389/frai.2026.1934450

**Published:** 2026-10-28

**Authors:** Chandni S. Sharma, Kathiravan Srinivasan

**Affiliations:** School of Computer Science and Engineering, Vellore Institute of Technology, Vellore, India

**Keywords:** artificial intelligence, body data privacy, bra fit, business governance, comfort measurement, intimate apparel, size recommendation, virtual try-on

## Abstract

Artificial intelligence (AI) for intimate apparel supports body measurement, size and product recommendation, garment simulation, and virtual try-on, but these tasks provide different evidence about fit and comfort. This critical narrative review examines direct bra research alongside adjacent apparel technologies and identifies the validation needed for business use. Recent direct studies include machine-learning models of breast morphology, bra recommendation prototypes, finite-element contact models, dynamic scanning, pressure measurements, and longitudinal wearer research. They establish meaningful component-level progress, while leaving uncertainty about transfer across products and populations and about the causal effects of AI-assisted decisions on customer and financial outcomes. Two-dimensional image quality, three-dimensional geometric agreement, physical support, perceived comfort, and business impact are therefore evaluated separately. An operational transfer test distinguishes methodological relevance from evidence of successful domain transfer. Building on the evidence gaps, the review proposes task-specific evaluation priorities, safeguards for intimate body data, and a staged research agenda with explicit resource and decision requirements. These proposals are analytical contributions, not empirically validated maturity models or deployment guarantees. The central implication is that businesses should match each proposed claim to its measured outcome and scale investment only after the relevant technical, wearer, and operational evidence is established.

## 1 Introduction

Intimate-apparel decisions combine nominal size, local body geometry, garment construction, support, and wearer preference. A correct size label does not establish comfortable pressure distribution, stable support during movement, or acceptable experience over a day of wear. Recent studies of breast-shape recognition and bra recommendations demonstrate concrete applications of artificial intelligence (AI), while physical and longitudinal studies show why comfort cannot be inferred from a label alone (Su et al., 2024; Sha et al., 2025; Paines et al., 2026). The business problem is consequently specific: choosing or designing a garment that meets a person's needs within a particular product range and use context.

AI contributes to different parts of this problem. Image analysis estimates landmarks or dimensions; tabular models classify or rank sizes; recommender systems combine product attributes and interaction histories; simulation estimates deformation or contact pressure; and generative models synthesize appearance. Reviews of fashion AI and recommendation document this methodological breadth (Chen et al., 2023; Deldjoo et al., 2024; Kouslis et al., 2025). These functions must be assessed against their own targets. A visually convincing garment image, an accurately classified morphology, and a comfortable garment are related achievements, but require different observations to verify.

The distinction has commercial consequences. A visualization service may improve product exploration without improving physical fit. A size assistant may help one brand while failing on another grading system. A pressure model may inform prototype design without supporting an immediate consumer recommendation. Research on strategic AI emphasizes matching a technology to a managerial task, and fashion-sector research links its use to organizational capabilities (Huang and Rust, 2021; Mohiuddin Babu et al., 2024). Neither establishes a general causal benefit for AI-based intimate-apparel retail.

This review has three connected aims: to critically assess published evidence on intimate-apparel fit and comfort; to examine which findings from adjacent technologies can inform that domain; and to derive a clearly labeled agenda for evaluation and business governance. Its contribution is the explicit connection between the scope of a technical claim, the evidence needed to support it, and the next feasible business or research decision.

The structure separates these aims. Section 2 discusses the review approach and evidence boundaries. Sections 3–5 examine concepts, data, and published technical evidence. Section 6 identifies gaps inferred from that evidence. Sections 7–8 present the authors' proposed evaluation framework and research agenda, followed by their limitations in Section 9. The discussion focuses on bras, including sports bras, because most of the direct studies examined concern those products. Extension to underwear, shapewear, or post-surgical garments requires additional product-specific evidence.

## 2 Review approach and evidence boundaries

### 2.1 Purpose and literature identification

This is a critical narrative Review using purposive sampling, not a systematic review. The databases used in the article search are IEEE Xplore, ScienceDirect, SpringerLink, Taylor & Francis Online, and MDPI. Eligible evidence comprised published studies of bra morphology, sizing, physical interaction, support, or wearer outcomes; published adjacent studies with a clearly identifiable computational task; and methodological or regulatory sources needed to interpret evaluation and governance. Sources were excluded from substantive evidence when they were consumer advertising, unrelated uses of ambiguous search terms, unsupported performance claims, or duplicates of a retained publication. Preprint-only discoveries were not used as evidence of established performance. Access limitations were recorded rather than treated as study defects. The bibliography identifies the retained sources; Tables 1–4 distinguish the main empirical evidence and technical resources. Reviews establish field context but are not counted as independent replications of the primary work they discuss.

**Table 1:** Direct computational and mechanical evidence.

Study	2D or 3D focus	Data and target	Reported contribution	Boundary of inference
Su et al. (2024)	3D scans to tabular features	201 female college students; breast morphology	Feature selection and morphology recognition; reported 99% recognition for three selected features	Study-specific morphology classes; recognition is not wearer comfort or retail recommendation success.
Sha et al. (2025)	3D scans to tabular features	150 individuals; six morphology clusters	Long short-term memory classifier and breast-to-bra mapping; reported 85.71% shape identification	Prototype and category accuracy; not a directly comparable endpoint to Su et al. or a causal business test.
Wang et al. (2025)	3D finite-element model	Adjustable bra contact; pressure and breast deformation	Mechanics-based analysis of body–bra interaction	Material, tissue, and contact assumptions condition the result; not an AI comfort predictor.
Han et al. (2026)	3D finite-element model	Sports-bra support and movement conditions	Simulated pressure and displacement compared with measurements	Findings are limited to the modeled bra support levels, movement speeds, and biomechanical conditions evaluated in the study.
Sang et al. (2026)	4D scanning and 2D patterns	Ten participants running at 8 km/h; motion and design	Dynamic surface capture connected to cup pattern and material choices	Small, activity-specific study; no general AI recommendation claim.

Direct denotes domain relevance, not evidence strength. Reported percentages use different labels and designs and are not a performance ranking. 2D, two-dimensional; 3D, three-dimensional; 4D, 3D geometry over time. AI, artificial intelligence.

**Table 2:** Direct wearer and bench evidence.

Study	Measurement focus	Design or population	Contribution	Boundary of inference
Keung et al. (2025)	3D scans and contact pressure	Seven athletic participants, bra size 75B; standing and cycling conditions	Auxetic and conventional pad comparison; localized pressure behavior	Narrow size range; pressure distribution alone does not establish universal perceived comfort.
Harbour et al. (2025)	Physiology and perceptual ratings	17 trained junior runners; three underband tightness conditions	Underband tightness associated with changes in respiratory and perceptual responses	Exercise-specific sample; support and physiological effects need separate assessment.
Paines et al. (2026)	Questionnaires, interviews and wear tests	Laboratory: 25 matched civilians; 14-week field study: 93 recruits enrolled, 56 final questionnaire respondents	Evidence across short laboratory tasks and sustained occupational wear	Military context and 40% loss to follow-up; longitudinal evidence, not AI-mediated retail value.
Maevskaia et al. (2026)	3D movement and mechanical testing	Configurable physical manikin	Repeatable comparison of bra support under controlled motion	Bench evidence; does not measure a wearer’s perceived comfort.

Direct sports-bra results remain conditional on the activity and population. AI, artificial intelligence.

**Table 3:** Comfort measurement options and interpretation.

Approach	Construct or use	Advantage	Limitation and proposed safeguard
Global rating with explicit anchors	Overall perceived comfort at a stated time	Low cost and suitable for repeated assessment	Can conceal local or thermal problems. Specify wording, anchors, context, and scale properties.
Local discomfort map and domain ratings	Cup edge, strap, underband, support, heat, wetness or movement	Separates locations and possible mechanisms	Multiple outcomes increase burden and selective-reporting risk. Prespecify primary outcomes.
Interface pressure and motion	Mechanical loading, stability and displacement	Localized physical measurements	Placement, calibration and motion affect readings. Use alongside self-report; physical measures do not replace perceived comfort.
Temperature and humidity	Thermal and moisture conditions near skin	Contextualizes thermal experience	Environment and sensor contact matter. Report conditions and use synchronized measurement windows.
Brief interviews or free text	Meaning, preference, dignity and omitted concerns	Identifies issues not captured by fixed scales	Interpretation requires a transparent coding approach; it does not produce interchangeable numerical labels.

The safeguards are proposals. No universal bra-comfort scale or physiological criterion standard is claimed.

**Table 4:** Recent virtual try-on methods.

Resource or study	2D or 3D focus	Task supported	Transfer permitted by the evidence
DeepFashion (Liu et al., 2016)	2D images	Recognition, retrieval and annotation	Useful visual representation; no bra mechanics or comfort ground truth.
SIZER (Tiwari et al., 2020)	3D scans and meshes	Size-sensitive clothing representation	Methodological relevance to garment geometry; casual garments do not establish bra fit.
SMPL (Loper et al., 2015)	3D body surface	Shape and pose representation	Initial body geometry; standard parameterization does not solve garment-induced compression.
VITON-HD (Choi et al., 2021)	2D images	High-resolution appearance synthesis	Appearance and alignment evidence, not validated support or pressure.
BooW-VTON (Zhang et al., 2025)	2D diffusion images	Mask-free synthesis in complex scenes	Evidence about visual rendering and input requirements.
VTON 360 (He et al., 2025)	3D-aware multiple 2D views	Consistent appearance from different viewpoints	Geometric consistency; not a constitutive garment–tissue model.
QuantFit-VTON (Nikolaev et al., 2026)	2D images plus measurements	Measurement-conditioned image geometry	Progress toward controllable visual size; no bra-specific comfort inference.
Clothing-size studies (Tan et al., 2024; Alhassawi et al., 2025)	3D anthropometry to tabular labels	General clothing-size prediction	Adjacent sizing methods; direct transfer requires matching garments and outcomes.

Dimensional focus identifies the representation and evaluated task, not an evidence-quality ranking. 2D, two-dimensional; 3D, three-dimensional; SMPL, skinned multi-person linear model.

### 2.2 Direct evidence and operational transfer assessment

Direct evidence concerns a bra, breast morphology relevant to bra design, or bra-related wearer experience. Sports bras are direct evidence for their stated activity and population, although transfer to everyday wear remains conditional. Adjacent evidence concerns general clothing, body reconstruction, image synthesis, or e-commerce. A general clothing-size study is not reclassified as direct merely because it includes torso measurements.

We propose a claim-specific transfer assessment using four questions: does the source predict the same quantity; are sensing and reference measurements comparable; are garment–body mechanics relevant to the proposed use; and is the measured endpoint the claimed outcome? For a proposed transfer, each answer should be recorded as supported, mismatched, or unreported. This is an analytical aid, not a validated scale. There is no compensatory three-of-four threshold: a mismatch in a necessary condition blocks that particular inference. A visual-synthesis result cannot establish pressure accuracy even when its inputs depict the same body region. A validated pressure simulator may support a mechanical hypothesis, but cannot establish perceived comfort without wearer evidence. A body-measurement algorithm can motivate a local validation study without proving successful cross-brand sizing.

The same source can therefore be useful for one claim and insufficient for another. General fashion ranking research is relevant to recommendation architecture; it does not establish a reduction in bra returns. This distinction is applied in the evidence tables and in the separation between Sections 3–5 and the proposals in Sections 7–8.

### 2.3 Analytical dimensions and safeguards against selective interpretation

The seven extraction dimensions defined in this review are: decision, inputs, target or reference outcome, model or method, evaluation design, deployment context, and possible business mechanism. They are informed by dataset documentation, model reporting, algorithmic auditing, and the National Institute of Standards and Technology (NIST) AI Risk Management Framework (Mitchell et al., 2019; Raji et al., 2020; Gebru et al., 2021; NIST, 2023). The first six dimensions organize what is observed or reported; the business mechanism is kept separate when it is a hypothesis. This is not an established seven-factor taxonomy derived from a validation study.

Selection bias cannot be eliminated by a narrative-review label. The safeguards used here are explicit scope boundaries, source-linked claims, inclusion of recent counterexamples to the initial gap statements, separation of direct and adjacent evidence, and disclosure when only an abstract was accessible. No numerical quality score is assigned from incomplete reports. Different tasks and outcomes are not pooled, and missing information is described as unreported rather than absent from the underlying study. These limits preclude estimates of how common a method or reporting practice is across the entire field.

## 3 Fit and comfort

### 3.1 Four distinct constructs

Size is a label within a particular grading system. Physical fit describes geometry and mechanics, including containment, ease, cup accommodation, band stability, strap load, contact pressure, and freedom of movement. Perceived comfort is a wearer's experience under stated conditions and duration; it includes tactile, thermal, moisture, movement, and psychosocial dimensions. Appearance concerns visual attributes such as coverage, silhouette, color, and style. Functional-textile literature treats comfort as multidimensional, while bra studies evaluate support and wearer experience through different measurements (Tadesse et al., 2021; Keung et al., 2025; Paines et al., 2026).

These distinctions determine the target. Measurement estimation is a regression task, morphology recognition is classification, product selection can be ranking, and repeated comfort ratings can be modeled as ordinal or continuous outcomes depending on the instrument. Image synthesis has an appearance target. The validity of one target does not automatically transfer to another: pressure is a mechanical measurement, not an objective replacement for a person's comfort rating. Similarly, retaining a purchased garment may reflect return costs or lack of alternatives rather than comfort.

### 3.2 Direct computational and mechanical studies

Recent direct studies extend beyond conceptual proposals. Su et al. (2024) used three-dimensional (3D) scans from 201 female college students to identify breast-morphology features. Sha et al. (2025) used data from 150 individuals to construct a breast-shape classifier and a mapping to bra recommendations. Their reported classification results concern their respective morphology definitions and study samples; they do not measure the same endpoint and should not be ranked against each other. Table 1 records these distinctions together with the physical modeling studies.

Finite-element research also addresses bra-specific interaction. Wang et al. (2025) modeled pressure and breast deformation in adjustable bras. Han et al. (2026) examined contact pressure and displacement across sports-bra support and movement conditions. These are direct mechanical studies, not evidence that a machine-learning model predicts individual comfort. Their contribution is to make physical quantities explicit and testable, while the interpretation remains bounded by the materials, bodies, boundary conditions, and comparison measurements used.

Dynamic acquisition adds information that static size labels omit. Sang et al. (2026) combined time-varying 3D scanning, material selection, and cup-pattern design in a study of ten participants running at 8 km/h. The work connects local deformation to a design process. It does not establish population-wide prediction or performance across unrestricted activities. Four-dimensional (4D) scanning denotes 3D geometry sampled over time; it should not be confused with two-dimensional (2D) image generation.

### 3.3 Wearer and experimental evidence

Table 2 summarizes direct wearer and bench evidence. Keung et al. (2025) compared auxetic and conventional pads using scanning and pressure measurements in seven participants with the same nominal bra size. This offers localized mechanical evidence under specific poses, with limited population coverage. Harbour et al. (2025) examined underband tightness in 17 trained junior runners and measured respiratory and perceptual responses. The study illustrates that a support decision may also affect another physiological or experiential outcome; tighter support cannot be treated as an unconditional improvement.

Direct longitudinal studies complement short-duration mechanical tests. Paines et al. (2026) combined questionnaires, interviews, laboratory trials, and a 14-week field study of military recruits. The laboratory study included 25 civilians matched to recruits, while the field study enrolled 93 recruits. Fifty-six recruits completed the final questionnaire; the reported 40% loss to follow-up was attributed to discharge or re-squadding. The findings demonstrate the value of extended wear observation, while attrition and the occupational setting limit their generalization to retail customers. Maevskaia et al. (2026) developed a configurable manikin for repeatable bra-support assessment. Such bench testing can isolate mechanical factors, but a manikin cannot report perceived comfort. These studies refine the central gap: the missing link is integration with externally validated AI decisions and attributable business outcomes, rather than a complete absence of direct or longitudinal bra research.

### 3.4 Comfort instruments and their interpretation

The instrument should match the aspect of comfort being studied. A global numerical, visual-analogue, or ordered-category rating is inexpensive and suitable for repeated use, but compresses several experiences into one value. Local discomfort maps can distinguish strap, underband, cup-edge, or underwire problems. Separate thermal, wetness, support, and movement ratings reveal mechanisms that a global score may conceal. Interviews and short free-text responses can identify concerns omitted from preset scales. Functional-textile reviews and the direct studies above use complementary subjective and physical approaches (Tadesse et al., 2021; Cheng et al., 2022). Table 3 compares their roles and limitations; it does not endorse a universally validated intimate-apparel comfort scale.

Pressure, temperature, humidity, and movement measurements can explain changes in self-report, but none is a universal criterion standard for comfort. Convergent evidence is useful; perfect agreement is neither expected nor necessary. A participant can prefer more support despite higher measured pressure, or report discomfort that a small set of sensors misses. Conversely, a pleasant short trial does not establish sustained suitability. The distinction matters for model labels: physiological signals should be used to characterize mechanisms, and self-report should remain the reference when the claimed outcome is perceived comfort.

## 4 Data and body representations

### 4.1 Anthropometry and the limitations of parametric models

Manual measurements, optical scans, and image-based reconstruction differ in acquisition cost, coverage, repeatability, and scale information. Reviews of body scanning and mobile measurement describe this range (Bartol et al., 2021; Idrees et al., 2024). Local landmark definition, posture, breathing, clothing occlusion, calibration, and capture instructions affect comparability. A low mean error across many body dimensions can coexist with substantial error in a dimension used by a particular size chart. The distinction between repeatability, agreement with a reference, and downstream usefulness is therefore necessary when interpreting a measurement result.

The Skinned Multi-Person Linear model (SMPL) represents body shape and pose through a learned mesh, blend shapes, and skinning (Loper et al., 2015). It is not a rigid-body model, and the original work also describes an extension for dynamic soft-tissue deformation. Nevertheless, the standard shape-and-pose representation does not provide a constitutive tissue model or solve garment-contact forces. Motion-dependent deformation learned from scans is not equivalent to predicting tissue compression caused by a particular band tension, cup, or material.

An estimated pre-contact body surface can be an initial geometry for a contact analysis; it is not by itself a validated prediction of the surface after loading or the associated pressure. The relevant extension could be a tissue-and-garment mechanics model or an empirically calibrated deformation model. Either requires comparison with observations for the intended body region and loading conditions. Bra-specific simulations illustrate why such modeling is relevant (Wang et al., 2025; Han et al., 2026), but do not establish one universally adequate representation. Diversity generated within a learned shape space also cannot guarantee coverage of morphology absent from its training data.

### 4.2 Garment properties and data resources

A body mesh alone does not determine garment fit. Pattern geometry, seams, elastic behavior, material direction, bending, component stiffness, and intended ease change the interaction. Work on fabric characterization and virtual ease allowance shows that these variables are part of the modeling problem (Power, 2013; Abtew et al., 2021). Garment measurements should be tied to a documented condition, such as relaxed or specified extension, rather than treated as interchangeable numbers. Manufacturing variation and wear-related changes can further affect the relation between a catalog specification and a delivered item.

Table 4 distinguishes the dimensional focus and evidential role of representative resources. DeepFashion provides imagery for recognition and retrieval; SIZER links 3D clothing representations to size; and virtual try-on datasets support appearance synthesis (Liu et al., 2016; Tiwari et al., 2020; Choi et al., 2021). These resources are valuable within their tasks. Their annotations do not automatically supply bra support, tissue compression, or contextual comfort labels. Likewise, general clothing-size prediction from 3D anthropometry remains adjacent evidence unless the tested garments and outcomes concern intimate apparel (Tan et al., 2024; Alhassawi et al., 2025).

Transaction and feedback data add information about choices, but their interpretation differs from a wear test. Stock, price, promotion, recommendation exposure, and return friction influence what is bought and observed. A return category should distinguish size, local fit, appearance, defect, delivery, and other reasons. The absence of a return cannot be treated as a verified positive fit label. Reviews of fashion recommenders distinguish user, item, and contextual information, reinforcing the need to preserve the provenance of each signal (Deldjoo et al., 2024).

## 5 Modeling approaches and decision support

### 5.1 Tabular models and bra recommendation prototypes

Classical classifiers, regression models, and ranking methods remain relevant when the inputs are measurements or garment attributes. General clothing studies evaluate machine-learning approaches using 3D anthropometry and size groupings (Tan et al., 2024; Alhassawi et al., 2025). Their practical attraction is compatibility with relatively structured data; their results still depend on the population, label construction, and sizing system. Architectural complexity is not evidence of better transportability.

Direct bra research now includes both feature-based morphology recognition and a long short-term memory network used in a recommendation prototype (Su et al., 2024; Sha et al., 2025). A morphology classifier answers which learned category an input resembles. The subsequent mapping from category to garment is a second step, requiring its own evaluation. Unless a study measures actual wearer outcomes, classification performance cannot establish recommendation satisfaction or comfort. When static measurement features are ordered as a sequence, the feature ordering and comparison with appropriate tabular baselines are also relevant to interpretation; the presence of a recurrent architecture does not make the observations longitudinal.

### 5.2 Image analysis and geometric estimation

Vision methods can estimate silhouettes, landmarks, body parameters, and measurements. Dense representations can be useful for correspondence or visualization, but prediction quality is affected by the relationship between the acquisition setting and the training data (Bartol et al., 2021; Idrees et al., 2024). An image without a reliable scale cue cannot uniquely determine absolute body dimensions from geometry alone. Learned priors can resolve an estimate statistically, but the resulting uncertainty must not be mistaken for a directly observed measurement.

The distinction between task outputs also applies to benchmarks. Reconstruction similarity may be useful for mesh fidelity while being insufficient for a particular band or cup dimension. A system that performs well on controlled capture may not preserve performance with loose clothing, mobile cameras, restricted mobility, or a different population. These are reasons to define the target and intended conditions; they are not evidence that every image-based system necessarily fails outside a laboratory.

### 5.3 Physics and hybrid models

Garment simulation makes some physical assumptions explicit. Historical work modeled dynamic garment pressure, and more recent bra studies examine localized contact and deformation (Zhang et al., 2002; Wang et al., 2025; Han et al., 2026). This gives physical models a different evidential role from an image generator: predicted pressure can be compared with instrumented pressure, and predicted displacement with measured displacement. Agreement remains conditional on material characterization, tissue representation, loading, and sensor accuracy.

Hybrid physics–AI models could approximate expensive simulations or estimate uncertain physical parameters, but this is a proposed research direction in the present review. A surrogate trained on simulated outputs inherits limitations in those outputs. A model should only be described as physics-informed when the physical relationship being enforced is stated, rather than when it merely receives a physical variable as input. The useful comparison is against both a data-only baseline and the underlying physical model, with real measurements reserved for evaluating the intended claim.

### 5.4 Recommendation and business evidence

Fashion recommendation includes item, outfit, and size tasks, with differing assumptions about users, products, and context (Deldjoo et al., 2024; Kim et al., 2024). Sheikh et al. (2019) described a deployed size-and-fit prediction system in general fashion e-commerce. This is relevant adjacent evidence that a computational prediction can be integrated into a commercial workflow. It is not a bra-specific estimate of customer benefit or profitability. Reviews of AI-enabled fashion transformation similarly provide organizational context rather than a causal estimate for intimate apparel (Mohiuddin Babu et al., 2024).

A product ranker must work within the available assortment and may inherit exposure or popularity bias from historical interactions. New users and products have limited histories, while model recommendations influence the data later used to train or evaluate the model. Offline ranking measures consequently address a narrower question than whether introducing the service improves customer experience. The reviewed sources do not support a pooled effect on intimate-apparel returns, margin, or satisfaction.

### 5.5 Generative virtual try-on and current developments

Virtual try-on research has advanced from improved high-resolution image alignment to diffusion-based handling of uncontrolled scenes and consistent multiple views (Choi et al., 2021; Islam et al., 2024; He et al., 2025; Zhang et al., 2025). BooW-VTON uses a mask-free training approach with pseudo-data to improve image synthesis under complex conditions. VTON 360 supports viewing-direction consistency through multiple 2D views and a 3D-aware representation. These advances address visual and geometric problems; they should not be dismissed simply because they do not solve garment mechanics.

Measurement-conditioned generation is a further development. QuantFit-VTON incorporates numerical person and garment measurements into diffusion-based generation (Nikolaev et al., 2026). Its relevance is controllable image geometry. The inference that such conditioning establishes bra pressure, support, or sustained comfort would still require independent measurements of those outcomes. Thus, the appropriate distinction is between different levels of fit-related evidence, rather than a blanket statement that all virtual try-on ignores size.

Language and multimodal interfaces may help retrieve catalog attributes, ask clarifying questions, or communicate validated results. In this review these are prospective implementation roles, not demonstrated intimate-apparel benefits. An explanation should trace to an actual measurement, product attribute, or model estimate. A generated statement that invents dimensions, material properties, or health implications cannot repair uncertainty in the underlying prediction.

## 6 Synthesis of evidence gaps

### 6.1 What is established and what remains conditional

The direct studies support component-level progress in morphology analysis, bra recommendation prototypes, mechanical simulation, dynamic design, and wearer assessment. The evidence does not justify calling the domain empty or uniformly immature. It instead reveals differences in what has been evaluated. A physically tested bra design and an accurate morphology classifier can both be useful while supplying different links in a decision process.

Our synthesis identifies three unresolved connections. First, the link from a measured or inferred body representation to a particular garment requires product-specific information. Second, the link from geometry or pressure to comfort requires wearer and context evidence. Third, the link from a recommendation to a business outcome requires a comparison that isolates the effect of introducing the decision service. These are logical evidence requirements derived from the claims, not prevalence estimates about all published work.

### 6.2 Comparability and external validity

The reported percentages in morphology recognition are not interchangeable with millimeter error, pressure agreement, ranking performance, or customer satisfaction. Different reference definitions, recruitment criteria, splits, and product populations also limit comparison within a nominal task. We therefore report study scope and target instead of presenting a leaderboard or pooled accuracy.

External validation is particularly relevant when a proposed use extends to another brand, size range, body distribution, device, or activity. The absence of such information in an accessible abstract is an uncertainty, not proof that the full study omitted it. Table 1 makes this access boundary explicit. A claim of broad deployment readiness would require more evidence than can be established from the limited descriptions available for several studies.

### 6.3 Evidence separation as the basis for the proposals

Figure 1 organizes the authors' interpretation of the evidence. It distinguishes observed component outputs from the proposed integration of those outputs into a business service. Its connections indicate evidence dependencies; they do not demonstrate interoperability or the existence of an end-to-end system. The evaluation, privacy, and management proposals that follow are offered as testable decision aids. None is a validated maturity scale or a guarantee of safer, more profitable deployment.

**Figure 1:**
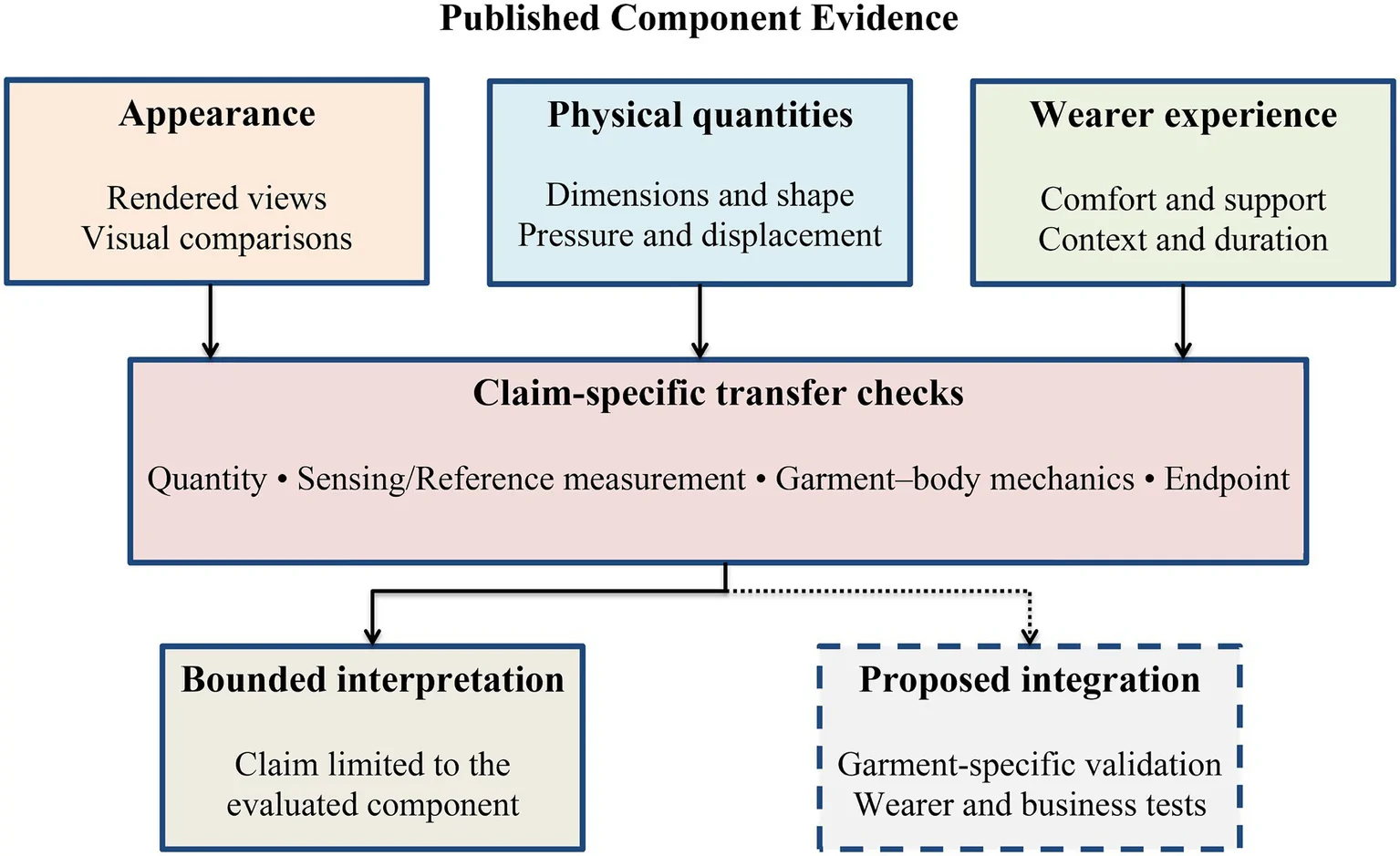
Evidence and proposed integration. Developed schematic distinguishing published component evidence from the additional validation required for a proposed integrated service. Solid connectors organize the review's interpretation; the dashed connector and box indicate proposed integration. Transfer checks are claim-specific and have no compensatory score. The schematic does not establish interoperability, implementation maturity, or validated end-to-end performance.

## 7 Proposed evaluation and governance framework

### 7.1 A feasible evaluation sequence

We propose a staged evaluation rather than requiring every study to fund a multimodal, multi-brand, longitudinal trial. Table 5 specifies the minimum evidence for different claims. A limited measurement study can make a limited measurement claim after checking reference quality, bias, error, and uncertainty under stated conditions. A claim about improved comfort needs wearer evidence. A claim about reduced returns needs operational comparison. The evidence budget should follow the claim's consequence and scope.

**Table 5:** Proposed evidence requirements matched to the claim.

Claim	Core evidence	Feasible initial design	Requirement before extending the claim
Body dimension estimate	Reference protocol; bias and absolute error in physical units; prediction-interval coverage and width	Limited-range study with participant-separated evaluation and a simple baseline	Test new capture conditions and intended populations; report failed measurements.
Size or product recommendation	Defined fit labels; class or ranking results; uncertainty	One product range, real alternatives and an assisted fallback	Test wearer decisions and additional products or brands; avoid substituting purchase for comfort.
Pressure or support prediction	Pressure or displacement agreement; material and loading assumptions	Instrumented prototypes or controlled bench study	Human validation for wearer claims; independent shapes and materials for generalization.
Perceived comfort	Repeated self-report; scale interpretation; context and duration	Brief wear study with optional sensor subsample	Longer wear or wider population testing when claimed; missingness and burden analysis.
Improved business outcome	Prespecified operational outcome, comparator and cost	Assisted pilot with exposure and reason-coded outcome logging	Controlled live evaluation with appropriate assignment, follow-up and causal assumptions.
Privacy or inclusion claim	Threat model or harm definition; stated privacy unit; subgroup uncertainty	Data-minimized workflow and targeted audit	Privacy-budget accounting for differential-privacy claims; sufficient intended-group evidence before broad assurances.

Each row supports only the stated claim. Timing, sample size and acceptable error require a local justification; initial designs do not automatically satisfy later-stage requirements.

At the first stage, define the intended decision, outcome, reference procedure, and acceptable error before choosing a model. Use a size chart, simple regression or classifier, or current human workflow as a baseline. Separate participants and related images between development and test data. Where the use case concerns unseen products, also hold out products; where it concerns an evolving service, use an appropriate temporal test. Holding out both users and products estimates a different, usually harder, scenario than testing existing catalog items on new users. The split should therefore be justified rather than imposed without reference to deployment.

For measurement, report dimension-specific mean absolute error, signed bias, and error quantiles in physical units. Report prediction-interval coverage as a proportion and interval width in physical units, with the nominal coverage level stated. For classification, show the confusion matrix and class-specific performance. For ranking, report relevance among available alternatives and the risk of unacceptable fit among top recommendations. A probability calibration measure should be paired with its definition, binning where relevant, and uncertainty; no single score establishes trustworthiness. Report failed captures, abstentions, and excluded cases in the denominator. A system that refuses difficult cases may appear accurate while serving fewer users.

Intervals and tests must reflect dependence. Multiple images, sensors, or wear sessions from the same person are not independent participants. Clustered resampling or a suitable hierarchical model can preserve that structure; crossed participant and product effects may both matter. Subgroup results need sample sizes and uncertainty, and sparse intersections should be described as inconclusive rather than fair. A pilot can estimate variability and recruitment feasibility before a precision- or power-based sample-size calculation. There is no universal minimum sample size that validates a fit system.

For comfort research, we propose brief repeated ratings with clearly stated anchors, body region, activity, and elapsed wear time. Instrument adaptation requires assessment of comprehensibility, reliability under stable conditions, and sensitivity to relevant changes; translating or shortening a scale is not automatic validation. Combine self-report and sensors through aligned time windows and prespecified hypotheses. Within-person changes should be separated from between-person differences, and composite scores should not be introduced without a defensible measurement model. A sensor subsample can control cost and burden, provided its selection and missingness are reported.

Participant burden is part of validity. A short baseline, a small number of scheduled or event-triggered ratings, and optional instrumented sessions may be more sustainable than continuous monitoring for everyone. Report invitation, enrollment, completion, sensor failure, and withdrawal counts, along with reasons where participants choose to provide them. Compare relevant baseline characteristics of completers and non-completers. Complete-case analysis can be biased when discomfort or capture burden affects dropout; mixed models or imputation require stated missingness assumptions, and sensitivity analysis is needed when missingness may depend on unobserved experience. These are proposed design safeguards, not procedures retrospectively attributed to the reviewed studies.

### 7.2 Intimate body privacy and privacy utility trade-offs

A harm assessment should extend beyond direct identifiers. Body-shape features can contribute to person recognition in adjacent computer-vision research (Chen et al., 2021). Removing faces or names therefore cannot alone establish anonymity of detailed body records. In an intimate-apparel service, linkage with accounts, transactions, or externally available images may increase risk. Possible inference of pregnancy, surgery, or health status should be treated as a threat scenario, not as a claim that such inference is reliably achievable from every scan. Disclosure can also cause embarrassment, coercion, or dignity and body-image harms without successful identity matching.

We propose collecting only information necessary for the defined service, separating account identity from measurement records, limiting raw-image retention, controlling vendor access and secondary training, and offering a manual-measurement route. These measures address different risks: pseudonymization reduces routine linkage but does not guarantee anonymity; encryption protects transport and storage but not misuse by an authorized recipient. Consent wording should distinguish recommendation, research, image retention, and optional reuse. Private capture conditions, respectful language, correction rights, and a practical deletion process are part of the customer workflow.

Federated learning keeps raw training data distributed, but locality alone is not a formal privacy guarantee (McMahan et al., 2017). Model updates, logs, and the released model can remain attack surfaces. Secure aggregation, update controls, and a threat model may therefore be needed depending on who is trusted and what is released. On-device inference similarly reduces some transmission while leaving local storage, backups, and model behavior to be protected.

Differential privacy bounds the change in an output distribution when a defined unit of data is changed (Dwork and Roth, 2014). The privacy unit must be explicit: protecting one record is weaker than protecting a person's entire set of repeated scans when that person contributes many records. The parameters *ε* and *δ* describe the formal guarantee; *ε* is not itself the noise magnitude, and neither parameter is a direct probability of re-identification. Sensitivity, clipping, mechanism, sample size, training procedure, and composition determine the required randomization and its consequences.

For a fixed mechanism and analysis, stronger privacy commonly requires more randomization or a more restrictive training budget. However, there is no universal mapping from *ε* to millimeters of sizing error, and no domain-specific threshold is established by the sources used in this review. Noise applied directly to a measurement is different from noise applied to gradients or an aggregate. A useful comparison would report the privacy unit, *ε*, *δ*, accounting procedure, clipping and noise settings, alongside dimension-level error, interval coverage, recommendation changes, and subgroup utility against a non-private baseline. Product-specific tolerances must be justified from garment and wearer evidence. Formal privacy also does not prevent all sensitive inferences based on population-level patterns.

### 7.3 Jurisdictional context and governance responsibilities

Table 6 summarizes selected statutory distinctions relevant to the proposed controls. Under the European Union (EU) General Data Protection Regulation (GDPR), identifiable body records are personal data, while the special-category rule for biometric data concerns processing for unique identification; health data have a separate basis for protection (European Parliament and Council, 2016). The EU Artificial Intelligence Act classifies uses by function and context, not simply by the presence of an AI model or body measurement; current implementation should be assessed using the applicable provisions and commencement dates (European Parliament and Council, 2024).

**Table 6:** Selected statutory distinctions for intimate body data.

Jurisdiction and source	Relevant distinction	Implication for the proposed workflow
European Union (European Parliament and Council, 2016; European Parliament and Council, 2024)	Identifiable body data are personal data. Biometric special-category processing concerns unique identification; health data are separately protected. AI regulation is use-specific.	Document purpose and actual processing. Assess the applicable data category, AI use and commencement provisions; do not infer a category solely from the word “body.”
California (California Legislature, 2026)	Sensitive personal information includes biometrics processed for unique identification and information collected and analyzed concerning health.	Evaluate covered-business scope and processing purpose. Link data controls and customer rights to the applicable provisions.
Illinois (Illinois General Assembly, 2008)	Biometric identifiers are enumerated; photographs and ordinary physical descriptions are excluded. Covered biometric processing has specific collection and retention rules.	Do not assume every torso measurement is covered by this biometric statute or exempt from other duties. Assess the actual capture and identification process.
China (National People's Congress, 2021)	Biometric and medical-health information can be sensitive; necessity, strict safeguards and separate consent requirements are explicit.	Assess the particular information, lawful processing conditions and any cross-border transfer before sharing body-derived records.
India (Government of India, 2023; Ministry of Electronics and Information Technology, 2025)	Digital personal data framework with consent and specified legitimate uses; no GDPR-style special-category list. Implementation is phased.	Distinguish enacted requirements from provisions currently commenced. Confirm operational dates and responsibilities for the actual service.

The 2025 Indian Rules commence different groups of provisions immediately, after 1 year, or after 18 months. This table is not a complete legal assessment; exceptions, territorial scope and amendments must be checked for implementation. AI, artificial intelligence; GDPR, General Data Protection Regulation.

United States state laws are not interchangeable. California's statutory definition includes biometric information processed for unique identification and personal information analyzed concerning health within sensitive personal information. Illinois' Biometric Information Privacy Act has a narrower enumerated definition of biometric identifiers and excludes photographs and ordinary physical descriptions (Illinois General Assembly, 2008; California Legislature, 2026). A torso scan should not automatically be classified as covered under every biometric statute. China's Personal Information Protection Law explicitly addresses biometric and medical-health information as sensitive and provides heightened necessity, protection, and consent requirements (National People's Congress, 2021).

India's Digital Personal Data Protection Act, 2023 addresses digital personal data and provides for consent and specified legitimate uses; it does not replicate the GDPR's special-category scheme (Government of India, 2023). Its implementation is phased. The 2025 Rules expressly stagger commencement, including later commencement for many operational provisions (Ministry of Electronics and Information Technology, 2025). The table is a scoped comparison for governance design, not a complete multinational compliance determination.

Figure 2 adapts the govern, map, measure, and manage functions of the NIST framework to a proposed intimate-apparel lifecycle (NIST, 2023). Responsibilities include a product owner for the permitted claim, apparel expertise for garment assumptions, technical ownership for validation and monitoring, and privacy and security responsibility for data access and incidents. Dataset documentation, model reports, and audit records can make these responsibilities inspectable (Mitchell et al., 2019; Raji et al., 2020; Gebru et al., 2021). Adoption of documentation alone does not establish legal compliance or prove that harms have been prevented.

**Figure 2:**
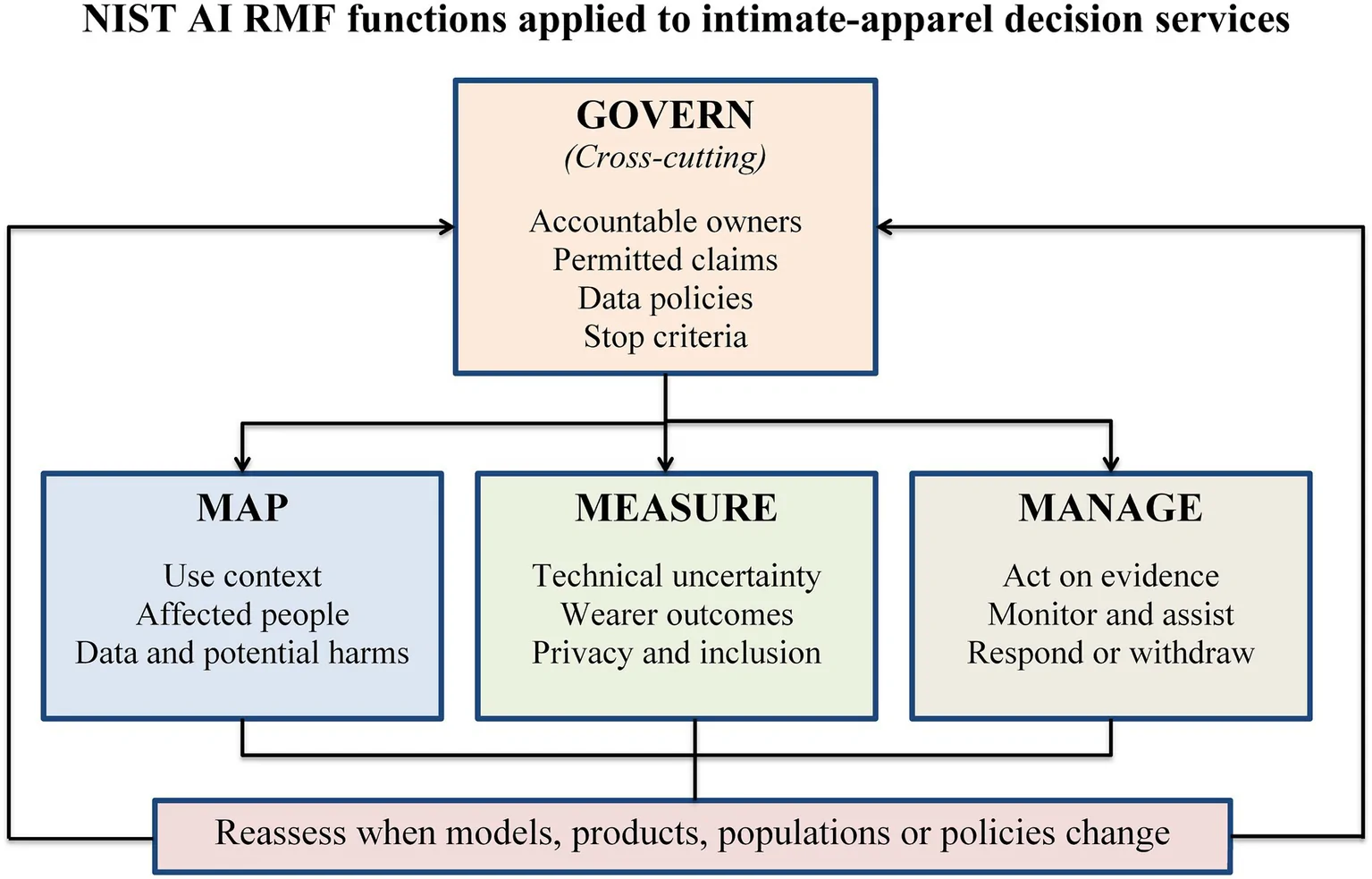
Proposed governance lifecycle. Developed application of the Govern, Map, Measure, and Manage functions of the NIST AI Risk Management Framework (NIST, 2023) to intimate-apparel decision services. Govern is cross-cutting, and the functions do not constitute a mandatory sequential algorithm. The displayed responsibilities and feedback loop are proposals for intimate-apparel services, not findings that governance adoption prevents harm or establishes legal compliance. NIST, National Institute of Standards and Technology.

### 7.4 Inclusion and user agency

The proposed fairness objective is to avoid unjustified differences in severe-misfit risk, measurement failure, recommendation coverage, and access to assistance. It should be defined with the use case and affected users, rather than selected solely because a metric is familiar (Ntoutsi et al., 2020). Product availability can be the limiting factor even when a model is accurate. A service should distinguish an unsupported body or garment range from a low-confidence prediction, and should not imply that the customer's body is at fault.

Collecting additional sensitive attributes for an audit creates its own obligations and must be justified. Where sufficient subgroup evidence cannot be obtained, narrower claims and assisted use are more defensible than an unsupported assurance of inclusion. Users should be able to correct measurements, decline images, compare alternatives, and contest or override a recommendation.

## 8 Proposed business hypotheses and research agenda

### 8.1 Testing the business mechanism

The business proposition is a hypothesis: more relevant assistance could change decisions, which could improve a process and eventually a customer or financial outcome. Figure 3 displays this proposed chain and the conditions that can interrupt it. It is not a measured causal model. The existing general-fashion deployment and strategic frameworks motivate investigation; they do not establish the chain for intimate apparel (Sheikh et al., 2019; Huang and Rust, 2021).

**Figure 3:**
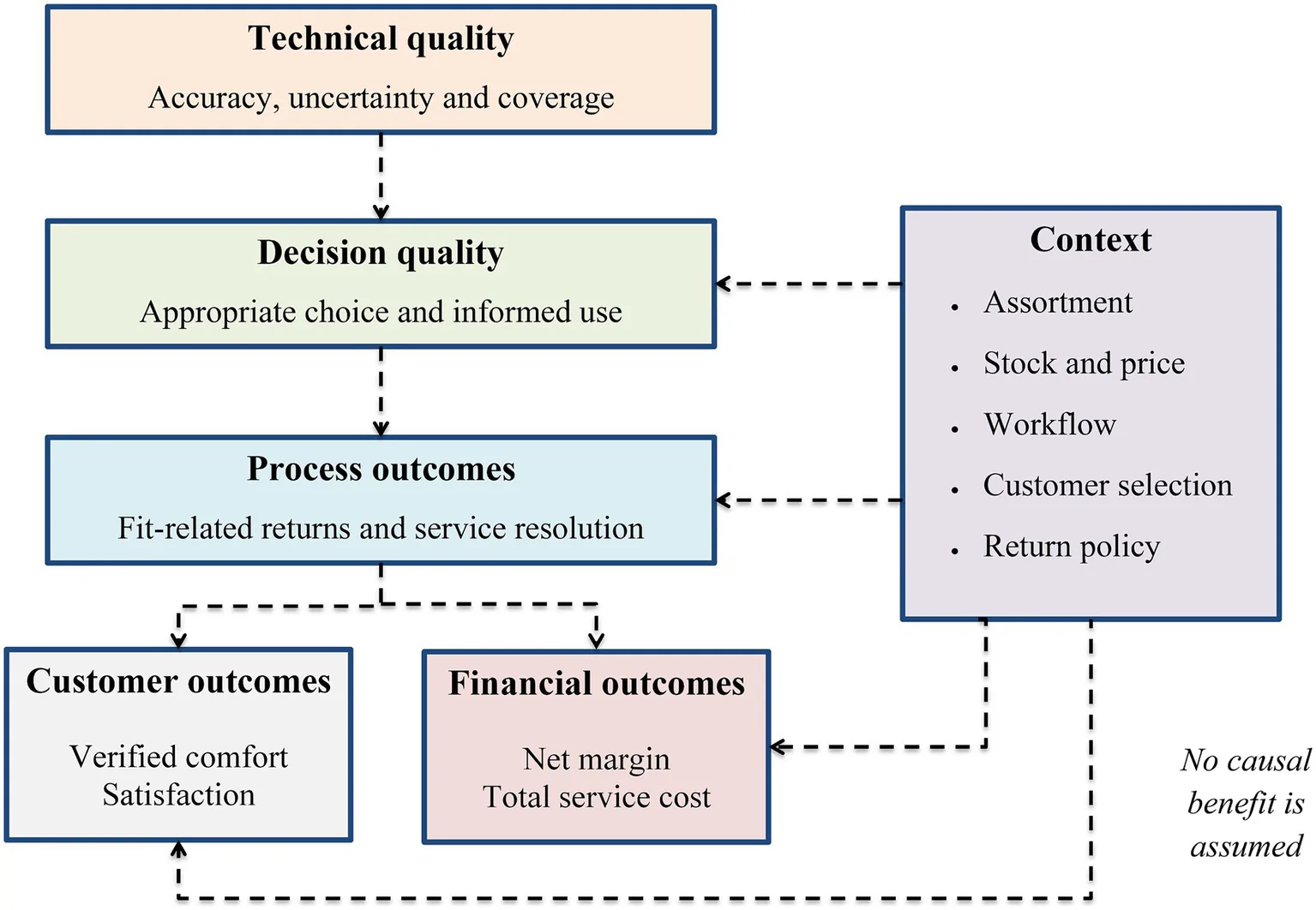
Business value hypothesis. Proposed links from technical quality to decision quality, process outcomes, and distinct customer and financial outcomes. Dashed connectors indicate hypotheses requiring separate empirical tests. Context may influence decision uptake, operational processes, and both customer and financial outcomes. The figure does not represent an estimated causal model or demonstrate that an intimate-apparel AI service reduces returns, improves satisfaction or comfort, or increases net margin.

A trial should compare a clearly specified service with the current size chart, quiz, fitter, or customer-service workflow. The primary outcome might be reason-coded fit returns, successful exchanges, service resolution, or verified comfort, depending on the intervention. Conversion alone is inadequate because a persuasive but inaccurate recommendation can move costs downstream. Financial analysis should include returns handling, support, acquisition, instrumentation, integration, inference, monitoring, and privacy or security controls. Satisfaction and dignity should be measured separately rather than inferred from revenue.

For a live service, randomized allocation at a suitable customer, store, or time unit can estimate the incremental effect of offering the intervention. Report assignment and uptake separately, allow for repeat customers and delayed returns, and specify stock and product eligibility. If randomization is infeasible, a quasi-experimental design needs an explicit identification assumption, diagnostics, and a discussion of concurrent changes. An interrupted time series without a credible comparison cannot automatically attribute a sales change to the model. Sustainability claims also require net accounting of logistics, product use, energy, and possible rebound, rather than assuming digitalization reduces impact (Ramos et al., 2023).

### 8.2 Sequencing work and allocating resources

Table 7 presents an illustrative staged program. Timings and person-months are planning assumptions proposed by the authors, not costs estimated from published trials or commitments by a funder. A person-month denotes one person's full-time effort for a month. Local wages, participant compensation, garment supply, approvals, infrastructure, and recruitment feasibility must be costed before adoption.

**Table 7:** Illustrative research phases and resource assumptions.

Phase and dependency	Work and evidence gate	Proposed staffing and resources	Potential funding owner
A. Months 0–6 Starting phase	Define claim, data dictionary, consent, reference measurements and baseline. Gate: credible labels and feasible recruitment.	6–10 person-months across apparel, data analysis, wearer research and privacy roles; secure storage, garments, compensation and basic measurement tools.	University–apparel partnership; institutional or public seed funding.
B. Months 6–18 after A	Test the next transfer condition and repeated comfort when claimed. Gate: uncertainty acceptable for the intended decision.	18–30 person-months; recruitment coordinator, apparel specialist, analyst and human-factors input; sensor subsample, calibration, participant follow-up.	Research grant with brand or testing-laboratory co-funding.
C. Months 18–30 after relevant B gate	Compare a live service with current practice. Gate: credible incremental benefit with acceptable harms and total cost.	12–24 person-months for integration, experimentation, customer service, analysis and monitoring; live traffic and delayed-return tracking.	Deploying retailer or brand with academic evaluation support.
D. Conditional months 24–36 or later	Pursue hybrid simulation or multi-party learning only if it resolves a measured bottleneck. Gate: benefit exceeds added complexity and risk.	24–48 person-months for specialist engineering and cross-partner coordination; instrumentation or secure distributed infrastructure as needed.	Consortium or externally funded research program with participating firms.

All durations and effort ranges are proposed planning scenarios. Person-months measure total effort. Local costing must add salaries, recruitment, garments, hardware, compute, security, and follow-up; phases may overlap or stop at a gate.

The first phase prioritizes an agreed data dictionary, claim, reference protocol, baseline, and consent process. It can begin within one institution and one product range. The second phase adds the next necessary source of evidence: a different capture setting or product range for transfer, or repeated wear for comfort. Instrumented benchmarks require equipment, calibration, and technical support. Basic, non-image measurements and brief ratings may provide a more feasible pilot, while still requiring careful recruitment and secure handling.

Later phases depend on the decision under test. Pressure or deformation research requires instrumented apparel expertise and a budget for material characterization and prototypes. Causal business research requires an operational partner, sufficient outcome volume, and access to reliable transaction and return records. Cross-organization privacy-preserving learning is appropriate only when the collaboration benefit, threat model, legal responsibilities, and harmonized labels justify its engineering cost. Its position in a program is conditional, not automatically the final stage of every deployment.

A university–apparel partnership could fund protocol development through an institutional or public research grant. An apparel firm or laboratory could co-fund wear trials and product testing. A retailer or brand deploying the service would normally need to fund integration, controlled evaluation, and monitoring.

### 8.3 Decision gates for scaling and procurement

A phase should advance when its prespecified evidence requirement is met. Early stopping is appropriate when the reference measurement is unreliable, recruitment excludes important intended users, privacy costs exceed the service need, or a baseline already meets the decision requirement. A result that is inconclusive because of sample size should lead to revised scope or further evidence, not a declaration of equivalence or fairness.

Build-or-buy decisions can use the same gates. A vendor should identify the tested populations, garments, intended claim, data rights, inference conditions, update process, and available audit outputs. Local validation should assess the customer's actual product range and workflow. Contracts and deployment plans should allocate responsibility for model changes, data deletion, incidents, fallback service, and termination. These are proposed procurement questions derived from the evidence gaps and generic audit principles (Raji et al., 2020; NIST, 2023), rather than a claim that one checklist guarantees business value.

## 9 Discussion

### 9.1 Contributions and competing priorities

This synthesis recognizes meaningful direct progress while resisting substitution between endpoints. Direct bra biomechanics and wearer studies are essential to AI evaluation because they help define what should be predicted. Adjacent technologies offer reusable methods and representations, but their evidential transfer is limited by the target, acquisition conditions, mechanics, and outcome. This distinction provides a more precise basis for business investment than either broad optimism about AI or a blanket characterization of the field as immature.

Several priorities can conflict. Detailed body data may improve a particular model while increasing exposure and participation burden. Additional support may alter pressure or other aspects of experience. Standardized protocols improve comparison while personalized comfort depends on context and preference. A richer model may be less useful than a simpler workflow if its data demands reduce participation or its uncertainty is concealed. These tensions call for explicit comparison of alternatives rather than a presumption that more sensing, more automation, or a larger model is preferable.

The proposed framework is intended to support such comparisons. It locates the evidence needed for each claim and makes decision consequences visible. It does not demonstrate a new algorithm, a validated psychometric instrument, a universal privacy budget, or a causal business benefit. Testing those propositions remains work for appropriately designed primary studies.

### 9.2 Limitations

Some direct studies were accessible only through abstracts; conclusions from them are restricted to what those sources report. Sample sizes and percentages are attributed to individual studies and are not reanalyzed as a shared dataset.

The direct evidence is concentrated in bras, and several studies use narrow participant or activity ranges. It should not be generalized to all genders, ages, body characteristics, intimate garments, or post-surgical needs. The transfer assessment and seven analytical dimensions are author-defined organizing devices, not validated instruments. No independent duplicate screening, formal risk-of-bias appraisal of the complete literature, or meta-analysis was performed. These limitations qualify both the gap synthesis and the resulting agenda.

Regulatory sources provide selected statutory context. Applicability depends on the actual data, purpose, geography, and commencement provisions. The research resourcing scenarios are illustrative and must be recalculated locally. These boundaries preserve the distinction between a critical review of published work and empirical evidence that a proposed service is ready to deploy.

## 10 Conclusion

AI for intimate-apparel fit and comfort has a substantive component-level evidence base, including recent direct bra research. Its outputs remain heterogeneous: morphology recognition, measurement, image generation, mechanical prediction, and perceived comfort are not equivalent endpoints. The central unresolved issue is how to validate the connections between these outputs and product-specific wearer and business decisions. This review proposes evaluating those connections in stages, beginning with a clearly defined claim, an appropriate reference, and a feasible comparison. Privacy, inclusion, and user agency belong within that evaluation because they affect both participation and consequences. Scaling an intervention should depend on the evidence relevant to its claim. The proposed diagrams and agenda make those dependencies explicit, while remaining hypotheses and decision aids for future validation.
